# Big data versus small data analysis towards personalized medicine practice

**DOI:** 10.1186/1878-5085-5-S1-A52

**Published:** 2014-02-11

**Authors:** Mira Marcus-Kalish

**Affiliations:** 1 Tel Aviv University, Israel

## 

The new era in medical research and development, based on new approaches and advanced technologies, enabled profound insights, in various levels and degrees of sensitivity, on the human body physical and mental functioning. The identification of these micro and macro environmental factors created a real need for new analysis tools and translational models. The "big data" created and harvested in health care, from the molecular to the physiological, imaging and environmental data, etc, created an inevitable growing need for sophisticated analysis tools. "In 10 years each individual will be surrounded by a virtual cloud of billions of data points—P4 medicine" 1. More than that there is a need to provide a broad understanding on the all factors interplay, based on combining all relevant data sources and utilizing all available analysis tools. New technologies are developed to harvest, verify, control, transfer, align and transform data, as well as new innovative analysis and predictive targeted tools to meet these challenges, both in academia and industry. In parallel there is a growing need to new innovative and sophisticated tools to analyse "small data". That is to enable reliable and responsible preventive, predictive and personalized medicine, in most cases where there is more data than equivalent records. New philosophical approaches, as well as innovative analysis tools, specially developed to meet these challenges, must be taken, based on the coupling of computer sciences, complexity theory, non linear dynamics, logic theory, etc. That is in compliance and in parallel to the statistical models such as generalized linear models or classification trees that are further endowed by new regularization methods, as well as recent developments in FDR (False Discovery Rate) testing emphasizing the hierarchical structure.

A targeted data mining tools will be presented to comply with the real need to deal with all interplay of all factors originated in different sources, such as: molecular, genetics, images, physiology, etc, presented in different scales. More than that the goal is to analyze small sets of data containing many parameters, including missing values. The presented patented rule discovery and prediction data mining tool, enables the simultaneous analysis of multilevel, multisource data (imaging, signals, categorical, numerical and descriptive data) as is, with no data manipulation, such as normalization, or neglecting part of the data. The goal is to relate to the whole data set as is, while handling missing data - which is one of the major needs in providing the “whole system” approach. The algorithm reveals the underlying rules while attaching a level of confidence to each rule, identifies the unexpected rules and detects the unexpected cases. Furthermore the tool was found to be less sensitive to over- fitting (small sets with many parameters).

In summary the Data Mining and prediction tool is proven to reveal *all If Than* rules, *all If than Not* rules and a subset of *If and Only If* (necessary and sufficient conditions) tools, with no prior assumptions. Three detailed examples will be presented. The evaluation of the Epoetin adverse effects to assess long term risks and advancing towards better Epoetin driven treatment modalities, based on the survival analysis of dialysis patients and cardiovascular patients treated with EPO. The second example will deal with dementia and neurodegenerative combined data taken from the hospital practice (MCI, AD and normal patients) to evaluate and optimize the various treatments and provide accurate predictions. The third example deals with the analysis of the progression of disease in ALS patients based on the patient’s current disease status. The data mining tool was already successfully applied to other applications such as: autoimmune diseases, drug development, personalized skin treatment, skin genetic data and biomarkers analysis, etc.

## References

[B1] Hood L The methods of fractal analysis of diagnostic images. Initial clinical experience Nat Biotechnol 2011 29 3 19121390010

